# Editorial: Plant cell wall in pathogenesis, parasitism and symbiosis, Volume II

**DOI:** 10.3389/fpls.2023.1230438

**Published:** 2023-06-20

**Authors:** Maïté Vicré, Vincenzo Lionetti

**Affiliations:** ^1^ Univ Rouen Normandie, Laboratoire Glyco-MEV UR 4358, Rouen, France; ^2^ Dipartimento di Biologia e Biotecnologie “Charles Darwin”, Sapienza Università di Roma, Rome, Italy; ^3^ Centro di Ricerca per le Scienze applicate alla Protezione dell’Ambiente e dei Beni Culturali (CIABC), Sapienza Università di Roma, Rome, Italy

**Keywords:** cell wall remodeling, symbiosis, cell wall integrity, plant immunity, plant parasitism, plant cell wall

A wide range of organisms that interact with plants must interface with the plant cell wall (CW) (Lionetti and Metraux, 2014). The view of the CW as only a static cellular barrier in these interactions is outdated. Cell wall polysaccharides, phenolic compounds, and proteins, in addition to regulating important growth and development processes, are also sources of elicitors that activate cell signaling pathways (Nguema-Ona et al., 2013). Surveillance mechanisms detect CW contacts with other organisms, and specific signaling pathways and responses are activated (Swaminathan et al., 2022) (**Figure 1**). During biotic interactions and abiotic stresses, the structure and composition of plant CW can be regulated at the biosynthetic level and through precise, continuous post-synthetic remodeling. As a consequence, the CW must be understood as a strategic space between organisms where intelligent and dynamic molecular strategies are implemented to overwhelm a fight or cooperate for specific physiological processes (Bacete et al., 2018; Castilleux et al., 2018; De Lorenzo et al., 2019).

**Figure 1:**
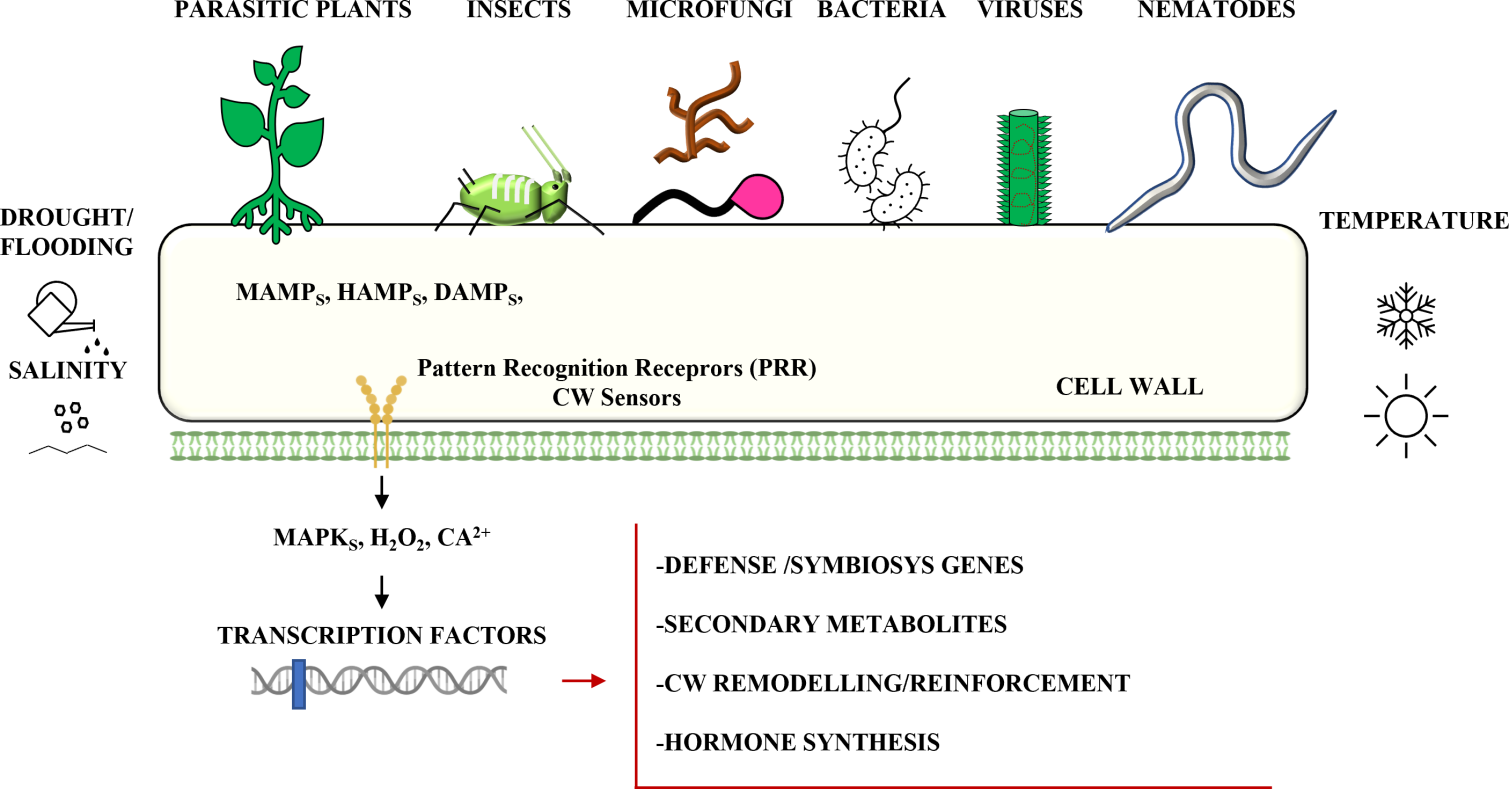
Schematic representation of the main systems of perception and responses related to the plant cell wall (CW) in the different biotic interactions. CW can play a central role in combined biotic and abiotic stress. Symbiont-induced CW modifications can improve plant development, nutrition, and tolerance to abiotic stresses. Abiotic stresses can alter CW composition, affecting efficient parasitism. M/H/DAMPS=Microbe/Herbivore/Damage Associated Molecular Patterns.

Cell wall enzymes and their inhibitors play key roles in apoplastic metabolism (Rui and Dinneny, 2020). Invertases (INVs) and pectin methylesterases (PMEs) play essential roles in carbohydrate metabolism, stress responses, and sugar signaling (Bellincampi et al., 2014; Tauzin and Giardina, 2014; Del Corpo et al., 2020). In this collection, Coculo and Lionetti reviewed the roles of invertase inhibitors (INVI) and pectin methylesterase inhibitors (PMEI) belonging to the “Plant Invertase/Pectin Methylesterase Inhibitor Superfamily” (Lionetti et al., 2017). An updated overview of the specific activity of the characterized isoforms, their specific functions in plant physiology, and their applications in biotechnology is provided. After the pioneering work in 2007 (Lionetti et al., 2007), several piece of evidence supported the role of PMEIs in plant resistance to stresses (An et al., 2008; Liu et al., 2018). With a genome-wide analysis and transcriptomics of the *PMEI* genes in *Brassica napus*, Wang et al. identified several *BnPMEIs* as resistance gene candidates in response to *Sclerotinia sclerotiorum*, suggesting them as possible tools to breed new and improved genotypes more resistant to *Sclerotinia* stem rot.

Specific CW changes can occur in the vicinity of plasmodesmata during viral infection (Lionetti et al., 2015; Stavolone and Lionetti, 2017). Viruses can modify pectin, callose, and structural proteins near plasmodesmata by increasing their size exclusion limit (Kozieł et al., 2021). López-González et al. addressed the little-explored impact of virus infections on secondary CW and plant development. The authors found a correlation between the developmental alterations induced in Arabidopsis by specific strains of turnip mosaic virus and specific changes in xylan and lignin biosynthesis. Although callose deposition is a response to both elicitors and pathogens, the mechanisms involved in its biosynthesis and degradation remain to be unraveled (German et al., 2023). Li et al. summarized the research progress on plant callose and its synthesizing enzymes in plant physiology.

Microfungi are also important etiological agents for plants (Doehlemann et al., 2017). The fungal pathogen *Sphaerulina musiva* causes stem canker with the consequent mortality of Populus trees. *Populus deltoides* can induce a lignified periderm to contain the pathogen, but the precise characterization of lignin changes in response to *S. musiva* infection is unknown. Bryant et al.identified a higher syringyl:guaiacil ratio, a higher Klason lignin content and lower p-hydroxybenzoate content in Septoria-infected *P. deltoides* trees compared to the healthy plant. This knowledge can favor biotechnological approaches aimed at improving the resilience and increasing the biomass yield of Populus for biofuel production.

Interesting contributions in this collection concern plant-parasite interactions. A fine-tuned re-arrangement of host CW is induced in response to infection by both plant-parasitic cyst nematodes and root-knot nematodes (Zhang et al., 2017; Bozbuga et al., 2018; Meidani et al., 2019). Veronico et al. found that drought stress affects CW metabolism in tomato roots, limiting feeding site development and reproduction of the nematode *Meloidogyne incognita*. Parasitic plants, such as Cuscuta species, severely damage economically important crops (Jhu and Sinha, 2022). These green parasites absorb resources through an invasive organ called the haustorium, which differentiates into vascular hyphae that establish a connection with the host plant’s vasculature. The degradation and modification of host CWs allow haustorium to effectively invade host tissues. Yokoyama et al. propose that *Cuscuta campestris* APETALA2/ETHYLENE RESPONSE FACTORS (ERFs) can activate the transcription of the CW enzymatic genes in haustorium to favor its invasion of tobacco and *Arabidopsis* plants.

Plant CW is a field of molecular dialogues and agreements with symbiotic microbes to establish intimate interfaces for developmental coordination and nutrient exchange (Balestrini and Bonfante, 2014). Plants can establish mutualistic symbiosis with arbuscular mycorrhizal fungi, and phosphorus transfer across the CW specialized interfacial compartment is an important process in the mycorrhizal pathway (Begum et al., 2019). Exploiting the *Rhizophagus irregularis-Lotus japonicus* interaction, Nguyen and Saito showed that polyphosphate in fungal CWs and apoplastic phosphatases play an important role in phosphorus transfer at the symbiotic interface in arbuscules. Mycorrhizal fungi can be involved in mutualistic interactions during orchid seed germination (Põlme et al., 2018). Chen et al. identified several genes codifying CW structural proteins such as epidermis-specific secreted glycoprotein, proline-rich receptor-like protein, and leucine-rich repeat (LRR) extensin-like protein, which are particularly involved in the symbiosis of Tulasnella and Serendipita fungi with *Dendrobium officinale.*

Bioinoculants represent an environmentally-friendly agricultural practice to alleviate drought stress in crops (Kour et al., 2022). The work presented by Wilmowitz et al. indicates that the inoculation of maize seeds with *Glomus* sp. and *Bacillus* sp. can help to cope with drought stress, preventing inhibition of photosynthesis and disruption of redox balance. *Glomus* sp. and *Bacillus* sp. can modify pectin methylesterification and hemicellulose content of maize leaves, possibly leading to alleviation of the negative effects of drought.

These important contributions advance our understanding of the relationships between plants and the environment at the CW interface, which will be helpful to engineer biotechnological strategies for agriculture and bioenergy fields. As a closing remark, we are grateful to the authors and reviewers for their invaluable contributions to this Research Topic.

## Author contributions

VL draft the editorial text. MV revised and approved the final version of the editorial. All authors approved the submitted version.
